# Design of Self-Integrating Transient Surface Current Density Sensor Integrated Fiber Transmission Link

**DOI:** 10.3390/s23177356

**Published:** 2023-08-23

**Authors:** Yifei Liu, Wei Wu, Xin Nie, Mo Zhao, Jiaqi Wang, Feng Wei, Wenbing Wang, Jinxi Li

**Affiliations:** 1 National Key Laboratory of Intense Pulsed Radiation Simulation and Effect, Northwest Institute of Nuclear Technology, Xi’an 710024, China; liuyifei@nint.ac.cn (Y.L.); wuwei@nint.ac.cn (W.W.); niexin@nint.ac.cn (X.N.); zhaomo@nint.ac.cn (M.Z.); wangwenbing@nint.ac.cn (W.W.); lijinxi@nint.ac.cn (J.L.); 2 National Key Laboratory of Antennas and Microwave Technology, Xidian University, Xi’an 710071, China; fwei@mail.xidian.edu.cn

**Keywords:** surface current density, high altitude electromagnetic pulse, split-gap shield loop (SSL), passive integrator, optical fiber transmission

## Abstract

The transient surface current density reflects the external coupling of the electromagnetic pulse (EMP) to the tested device. In this paper, the generation mechanism and measurement principle of conductor surface current density are introduced, and the surface current density distribution irradiated by EMP on a typical aircraft structure is simulated and analyzed. The traditional surface current density is usually measured by B-dot antenna, but its output signal is the differential of the measured signal, so additional integrators or numerical integration of the measured data are required. In this paper, a self-integrating surface current sensor based on optical fiber transmission is designed based on the shielded loop antenna with gap structure. The output signal is the real signal waveform to be measured. Compared with coaxial cables, integrated optical fiber transmission improves the anti-interference ability of long-distance transmission signals. At the same time, the design process of the sensor is introduced in detail. The bandwidth of the sensor is 300 kHz~500 MHz, the sensitivity is calibrated at 1.23 (A·m^−1^)/mV, and the dynamic range is ±25~1400 A·m^−1^ (35 dB). The surface current of a metal plate is measured in a bounded wave electromagnetic pulse simulator using a detector developed in this paper. The test results show that the developed sensor has good engineering applicability.

## 1. Introduction

High altitude electromagnetic pulse (HEMP) poses a great threat to electronic systems such as aircraft, missiles, satellites, etc. [1,2]. An electromagnetic pulse radiation sensitivity test of system-level equipment is generally carried out by electromagnetic pulse simulator, and based on the monitoring data of the effect parameters of electronic equipment (cable, antenna coupling current, etc.), the safety margin of the tested equipment meets the design requirements [3,4,5].

Under HEMP irradiation, the physical quantity of surface current reflects the external interaction between the tested equipment and HEMP, and the surface current further forms a scattering field, which is helpful for the electromagnetic environment analysis of ships and other integrated systems and the evaluation of radiation immunity performance of external exposed equipment [6,7]. In addition, the coupling field in the cavity formed by the leakage of holes and cracks in the shell is also related to the surface current density [8]. The ground condition of the equipment under test also affects the current distribution on the surface of the equipment shell. Therefore, it is of great significance to develop the surface current distribution of the equipment shell in the electromagnetic pulse effect test.

As early as the 1970s, the United States carried out theoretical research on the surface current density and charge density of aircraft under the action of plane waves. Based on the geometric diffraction theory, the surface current density and charge density distribution of B-1 and 747 aircrafts under the irradiation of continuous wave and transient electromagnetic pulse were given, and the surface current density measurement was measured by B-dot sensors in tests [9,10]. A surface current injection technique (SCIT) was proposed and a mobile injection source simulator was developed in order to detect the hardening performance of aircraft against HEMP relatively simply [11]. MGL B-dot Sensors, manufactured by EG&G in the United States, provided a strong support for the relevant measurement [12]. In China, Zhang of Southeast University designed a surface current sensor based on semi-circular Rogowski Coil with an effective working bandwidth of 70 kHz to 195 MHz [13]. In recent years, with the rapid development of electromagnetic calculation, numerical simulation provides convenience for the study of the surface current distribution. In [14], based on an improved leapfrog alternating-direction (ADI) finite-difference time-domain (FDTD) method, the surface current distribution of complex structures under the action of electromagnetic pulse was calculated, and the surface current variation laws of aircraft and tank were given [15].

The measurement of the surface current density is essentially the measurement of the magnetic field signal. There are many ways to sense magnetic fields; in [16], a transient magnetic field sensor based on a digital integrator was developed. The antenna was a small B-dot loop, but it is not suitable for the measurement of the surface current density due to the large size of the detector. With the development of magnetic signal measurement technology, more and more kinds of magnetic sensors are being used, such as magnetoresistive sensors, optical fiber magnetic field sensors, refractive index sensors, and so on [17]. The performance of different kinds of sensors varies greatly. For example, the magnetoresistive sensor is suitable for the measurement of low frequency magnetic field [18], and the refractive index sensor based on supermaterial is a hot research topic in recent years, but its performance in terahertz frequency band is excellent [19,20]. In fact, there are many factors affecting the sensor measurement of surface current density, especially frequency response, size, and power, but the current more popular sensor technology will be the development direction of surface current density measurement.

Aiming at the measurement of the surface-induced current of the shell under high altitude electromagnetic pulse irradiation, this paper reviews the generation mechanism of Js, and takes a typical aircraft structure as the object, simulates and analyzes the distribution law of the surface-induced current, and obtains the index design basis of the surface current density detector. A self-integrating surface current sensor based on optical fiber transmission is developed to overcome the disadvantages of the traditional measurement techniques. The sensor is calibrated in a transverse electromagnetic (TEM) cell and the surface current density of a metal plate is measured under a bounded-wave EMP simulator. The performance of the detector is verified.

## 2. Theoretical and Simulation Analysis

When the electromagnetic wave propagating in free space irradiates a uniform medium, an induced current will be generated on the surface of the shell, and the induced current will further generate a new electromagnetic wave. The induction process can be explained by scattering theory [21]. Assuming that the electric field (E-field) and magnetic field (***H***-field) of the incident wave are ***E****_i_* and ***H****_i_*, respectively, and the medium is ideal conductor in which the internal field is zero, the scattered E-field and H-field excited by the medium are ***E****_s_* and ***H**_s_*, respectively. According to the principle of generalized equivalence, the conductor can be removed and induced current can be seen as existing on the boundary surface. According to the uniqueness theorem of the solution of Maxwell’s equations, once the outer surface of the conductor meets the boundary conditions of a tangential E-field or H-field, a unique solution can be obtained [22]:

(1) $$E_{s,t}+E_{i,t}=0,$$

(2) $$H_{s,t}+H_{i,t}=n\times J_{S},$$

The subscript *s* is the tangential component of the conductor surface, and ***n*** is the outward directed normal vector on the surface, and ***J****_s_* is the sum of conduction current density and displacement current density. For ideal good conductors, the displacement current can be ignored, and the surface current density is numerically equal to H-field intensity on the shell surface, and the two are orthogonal in direction.

**Figure 1:**
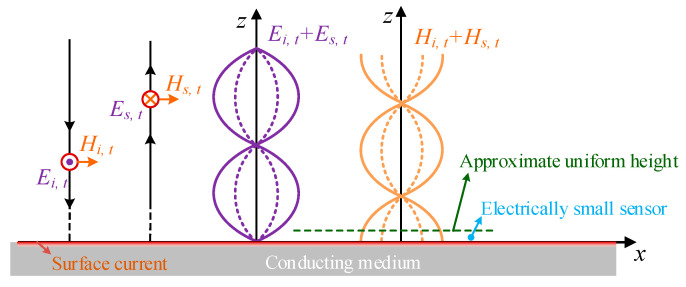
Time-harmonic electromagnetic wave irradiated infinite conductor plate model.

Figure 1 shows a model of time-harmonic electromagnetic wave irradiation on an infinite planar conductor. It is shown that the total reflection would occur in this ideal situation. Therefore, the scattered waves are total reflected waves. The composite of incident and reflected waves is a standing wave, with a phase difference of 90° between E-field and H-field. The antinodes of H-field appear at the interface [23]. Due to the infinite size of the conductor, the distribution of the surface current is uniform, meaning that H-field intensity at any point on the conductor surface is identical and can be approximately considered uniform within a certain height. The height is related to the frequency of the electromagnetic wave, and it decreases with increasing frequency. If an electrically small ***H***-field probe is placed on the surface of the conductor, the output of the detector characterizes the current density of the conductor surface.

The validity of using a single sensor or single-sided measurement for determining surface current density is based on the assumption that the magnetic flux cannot penetrate the medium. If a B-dot sensor (loop aerial) is used, in order to ensure the above assumptions are true, the skin depth of the conductor is at least three orders of magnitude smaller than the radius of the ring antenna. Furthermore, in order to apply the simplification hypothesis (*H* = *J*_s_), the conductor thickness must be greater than five times the skin depth [12].

When HEMP irradiates an electronic system, it is hard to accurately predict the distribution of its surface current. In order to analyze the surface current distribution on an equipment shell, a simplified airplane model under HEMP irradiation is calculated by CST. The HEMP is defined as follows [24]:

(3) $$E(t)=kE_{0}(e^{-\alpha t}-e^{-\beta t}),$$

where *E*_0_ = 50 kV/m, *k* = 1.3, *α* = 4.0 × 10^7^ s^−1^, β = 6.0 × 10^8^ s^−1^. The ***E***-field strength is 50 kV·m^−1^, and the ***H***-field strength is 132.5 A·m^−1^.

**Figure 2:**
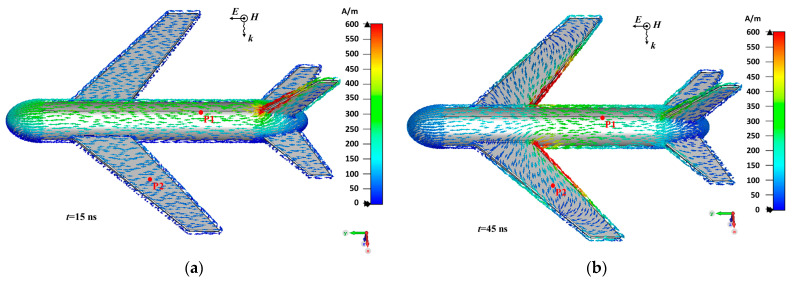
Induced current distribution on airplane surface: (**a**) time is 15 ns; (**b**) time is 45 ns.

The fuselage is approximately a circular cylindrical structure with a length of 13 m and a diameter of 1.8 m, while the wing is approximately a flat structure with a length of 7 m and a width of approximately 2 m (maximum width: 2.6 m, minimum width: 1.4 m). The simulation set HEMP to irradiate the upper surface of the aircraft vertically, with the direction of the electric field parallel to the fuselage. Figure 2 shows the surface current distribution with the simulation times of 15 ns and 45 ns, respectively. It can be seen that in the primary stage, under the force of E-field, induced current on the airplane surface remains consistent with the direction of E-field. Later, the induced current on the wing converges in the direction of the fuselage.

**Figure 3:**
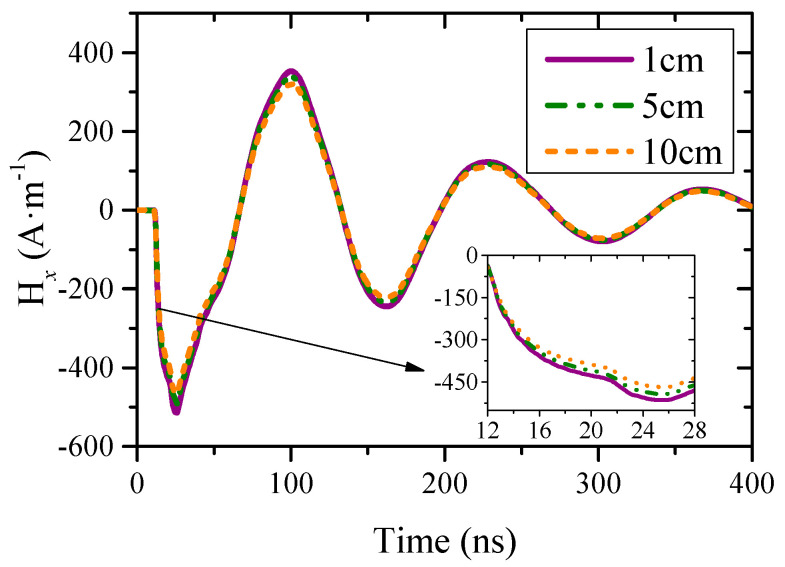
***H***-field waveforms above point P1 in *x*-direction.

Figure 3 shows the ***H***-field waveforms in x-direction above the point P1 on the fuselage, which represents the surface current density in *y*-direction. It can be seen that the surface current exhibits a single frequency attenuation oscillation waveform with a peak value of 500 A/m. This is due to the high energy coupling efficiency of the fine linear structure at its resonant frequency [25]. The approximate height of the ***H***-field uniform region is about 5 cm.

**Figure 4:**
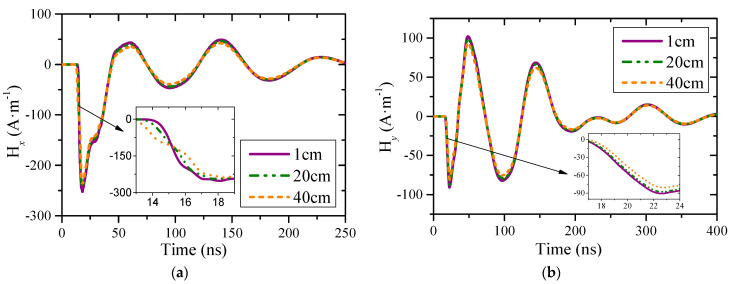
***H***-field waveforms above point P2: (**a**) in *x*-direction; (**b**) in *y*-direction.

Figure 4 shows the ***H***-field waveforms above the point P2 on the wing. It can be seen that the peak value in *x*- and *y*-direction are 250 A·m^−1^ and 100 A·m^−1^, respectively. Both waveforms have steep falling edges because the coupling efficiency of the plate structure to sensitive frequency points is not outstanding, and the coupling efficiency in high frequency band is consistent. In addition, the approximate height of the ***H***-field uniform region above the wing is about 20 cm, which is better than the cylindrical fuselage.

The transient induced current on the airplane surface can be analyzed by Fourier transform into the frequency domain. If the surface current is uniform in a certain region, the height of the approximately uniform region of ***H***-field can be determined by the high-frequency component of the transient current.

However, when the surface current distribution is not uniform, the magnetic field above the surface of the shell will be affected by the surface current contribution of other positions, which would lead to the height of the field uniform region being reduced. Therefore, the sensor should be installed on a flat surface to ensure that the surface current density is approximately constant, and installation should be avoided on the surface of the sharply curved shell, and near the edge of the shell, the connection position, and the opening of the hole.

According to the simulation results, it can also be seen that due to the limited size of the real shell, the energy of HEMP in the low-frequency band is difficult to be induced, so *J_s_* detector’s low frequency performance can be slightly worse. However, due to the structural characteristics, the peak amplitude of the surface current density is much larger than that of the incident magnetic field, which also requires that the dynamic range of the designed sensor should be large enough.

## 3. Sensor Design and Calibration

As the measurement result of B-dot sensor is the differential signal of the ***H***-field, and the signal is usually transmitted over a long distance by the coaxial cable, this makes this type of sensor inconvenient in engineering applications [26,27]. If the output signal of a sensor is the true surface current density waveform and can be transmitted by a light-weight, anti-interference optical fiber [28,29], this is obviously more advantageous.

### 3.1. Sensor Design

**Figure 5:**
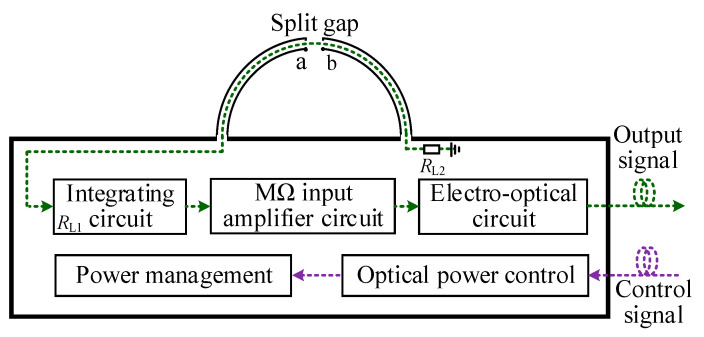
Design scheme of the proposed sensor.

As shown in Figure 5, the design scheme of a self-integrating sensor based on optical fiber transmission is presented. A semicircular Split Shielded Loop (SSL) is installed on the upper surface of the shield shell, and the hardware circuit is integrated in the shield.

In the practice of measuring the intensity for the magnetic component of an electromagnetic field, it has been proved that SSL has an advantage versus the unshielded loop antenna, because it can effectively suppress the interference of the electric field without differential balun [16]. In this design, SSL is made of semi-rigid coaxial cable (SR086-50) with characteristic impedance of 50 Ω. In order to ensure impedance matching, both terminal loads of the inner core are 50 Ω.

**Figure 6:**
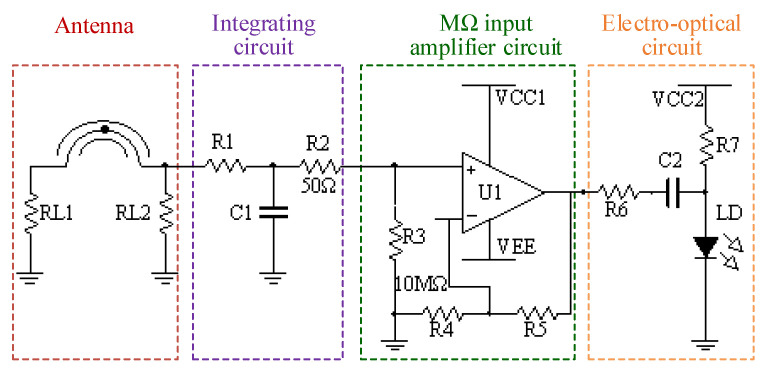
Schematic of the signal processing circuit.

The hardware circuit inside the shield is composed of a signal processing circuit and remote control circuit. In order to ensure that the sensor is electrically small, the volume of the shield should be designed as small as possible. The schematic of the signal processing circuit is shown in Figure 6. It is mainly composed of integrating circuit, MΩ input amplifier circuit, and electro-optic conversion circuit.

The integrating circuit is designed with passive RC circuit, which is used to recover the real waveform from the output signal of the SSL antenna. When the input signal of the integrating circuit is *V*_in_(*t*), the current-limiting resistor *R*_1_ charges the integration capacitor *C*_1_. If the impedance of *R*_1_ is much greater than the capacitive reactance of *C*_1_, the output signal of the circuit is:

(4) $$V_{\mathrm{out}}\left(t\right)=\frac{1}{R_{1}C_{1}}\int V_{\mathrm{in}}\left(t\right)dt,$$

For a single pulse signal, the time constant *τ* = *R*_1_·*C*_1_ should be greater than the duration of the pulse. The principle of the integrating circuit is simple, but it is difficult to realize an integrating circuit with excellent performance due to the stray parameters of the circuit. Theoretically, the parasitic inductance of capacitor *C*_1_ determines the high-frequency performance of the integrator. A more ideal match is that the resistor *R*_1_ takes a number of kΩ, and the capacitor *C*_1_ takes hundreds of pF to 1 nF.

The output of the passive integrator needs to be connected to a circuit with an input impedance much greater than *R*_1_ to collect the voltage on the integrating capacitor *C*_1_. In this design, an FET operational amplifier with input resistance of GΩ is selected as the core device. In order to avoid the abnormal operation of the amplifier caused by the floating of the positive input port, the resistor *R*_3_ of 10 MΩ is connected to the ground. Set the feedback resistor *R*_4_ equal to the gain-setting resistor *R*_5_, and the amplifier closed-loop gain is 2 V/V.

The resistor *R*_7_ in the electro-optic circuit provides a suitable bias current for the semiconductor laser. The output signal of the amplifier circuit drives the semiconductor laser based on amplitude modulation, so the tested signal is converted into an analogue optical signal and can be sent to the receiver module through a fiber optic cable.

The control circuit in the shield can receive different commands from the receiver module: (1) the sensor can output a self-detecting signal to check the link integrity and optical losses to ensure accurate measurement; (2) the sensor can remotely be put in power on/off status, or in a low power standby mode to save the battery power when not used.

**Figure 7:**
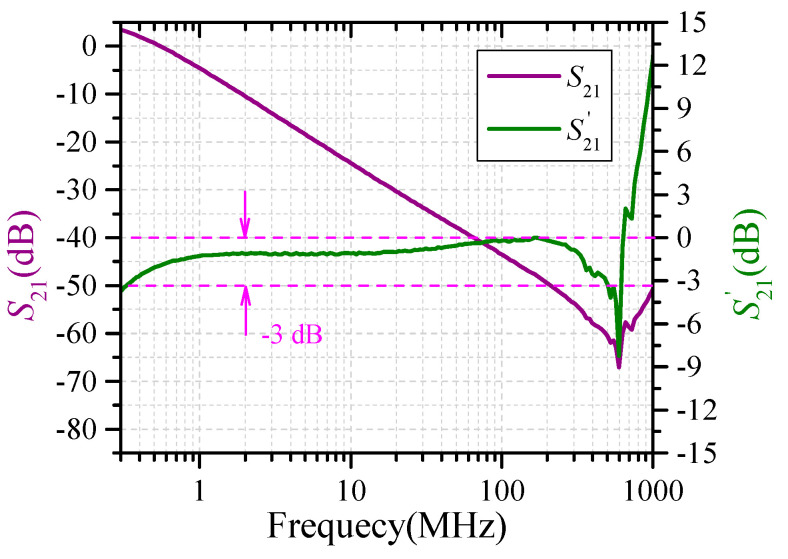
Frequency response of the signal processing circuit.

The frequency response of the signal processing circuit connected with optical receiver is measured by a vector network analyzer (VNA). The purple waveform in Figure 7 is the original measuring results. The downward-sloping straight line corresponds to the transmission bands of the system, and the center frequency is 600 MHz. Since the output of the circuit is inversely proportional to the angular frequency ω, in order to determine the −3 dB bandwidth of the circuit, the measured S_21_ curve is transformed according to the following formula:

(5) $$S_{21}^{\prime }=20\log_{10}\left(\frac{V_{\mathrm{out}}\left(f\right)•\omega }{V_{\mathrm{in}}\left(f\right)}\right)=S_{21}+20\log_{10}\left(\omega \right),$$

where *V*_in_(*f*) and *V*_out_(*f*) are the input and output voltages of the VNA with frequency. The olive curve in Figure 7 represents the transformed waveform ($S_{21}^{\prime }$ is added a constant), and it can be inferred from the curvet that the −3 dB bandwidth of the signal processing circuit is approximately 300 kHz~500 MHz.

**Figure 8:**
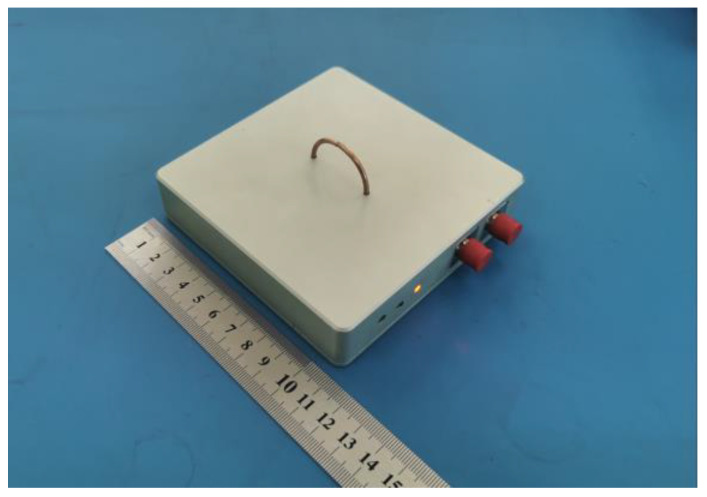
Photograph of the designed sensor.

The photo of the designed sensor is shown in Figure 8. It is battery powered and especially shielded for a high immunity to electromagnetic fields. The length and width of the shell are 11 cm and the height is 2.5 cm, respectively. The radius of the SSL antenna is 1.5 cm and the length is about 5 cm. In fact, SSL will have a parasitic inductance, and the inductance value will affect the high-frequency performance of the antenna, but the coaxial cable for making SSL is very short, and the antenna inductive reactance can be ignored within the −3 dB bandwidth of the signal processing circuit.

The shield shell of the sensor is no longer electrically small size with the increase in frequency. The simulation results based on CST show that the shield will form a resonance at 1.2 GHz. When the frequency is less than 800 MHz, the resonance effect can be ignored, so it can be regarded as electrically small size. Therefore, the working bandwidth of the detector can be defined to be 300 kHz~500 MHz.

### 3.2. Measurement System Calibration

**Figure 9:**
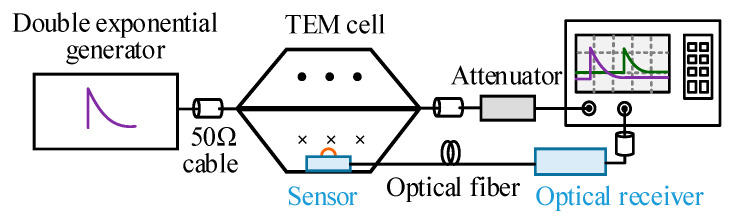
Schematic of the calibration system.

Since the surface current density detector is essentially a measurement of the magnetic field intensity, the calibration of the measurement system can be carried out in the TEM chamber, which can generate a uniform electromagnetic field environment [30,31]. As shown in Figure 9, the calibration system is constructed to test the double exponential wave response of the developed sensor.

The TEM cell is a deformed transmission line structure, and it transforms the double exponential pulse voltage V(*t*) into a homogeneous electromagnetic field. The magnetic field in the TEM cell is:

(6) $$H\left(t\right)=\frac{V\left(t\right)}{d•\eta },$$

where *d* is the distance between the septum and the ground plane of the TEM cell (*d* is 15 cm in the used TEM cell), and *η* is the impedance of free space wave, 377 Ω.

**Figure 10:**
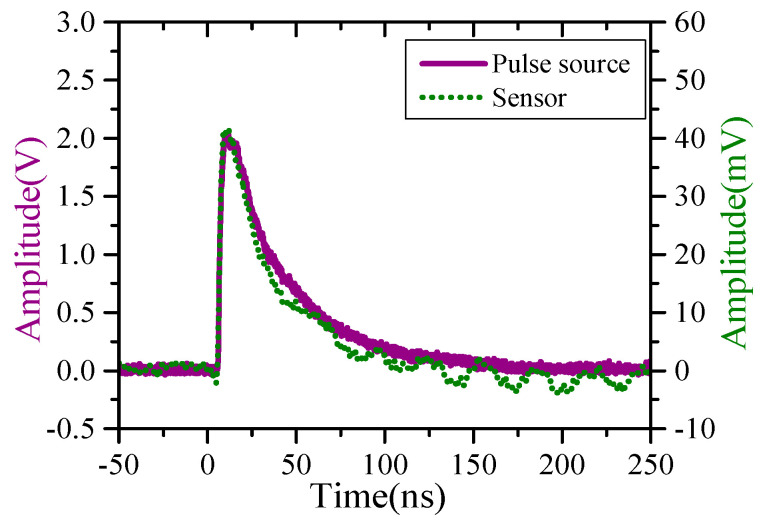
Typical calibration waveforms.

The pulse generator can output double exponential with rise time of 2.5 ns and half-height width of 23 ns. Figure 10 shows a typical calibration waveform. It can be seen that the output wave of the sensor is in good agreement with that of the pulse generator, except for a slight difference of the half-height width between them, which is mainly due to the insufficient of the low-frequency performance of the sensor. Considering the electric field component in the TEM cell, it shows that the detector has a strong ability to suppress the electric field interference.

The sensitivity coefficient of the sensor is *k* = 1.23 (A·m^−1^)/mV by linearly fitting multiple sets of calibration data. Due to the limited output value of the pulse generator, the saturation output value of the sensor is not obtained. We use an equivalent solution to inject a sinusoidal signal into the antenna port of the sensor, and the saturation output value of the system is about ±1.2 V. The peak-to-peak value of the background noise of the system is roughly 5 mV. According to the signal-to-noise ratio, the minimum detectable signal of the system can be specified as 20 mV. Thus, the dynamic range of the measurement system is ±25~1400 A·m^−1^ (35 dB).

## 4. Experimental Test

**Figure 11:**
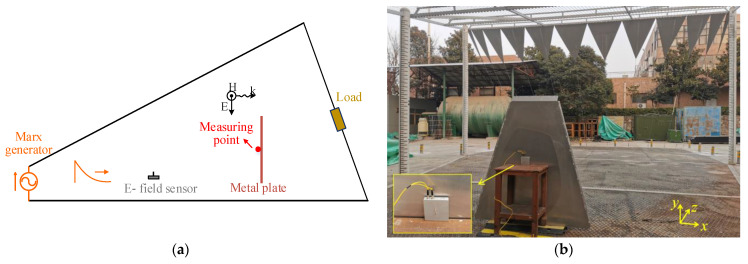
Experimental scheme and test photograph: (**a**) experimental scheme; (**b**) test photograph.

The performance of the sensor has been tested in Section 3.2. Furthermore, the surface current density of the metal plate is measured in bounded EMP simulator by using the developed sensor. The experimental scheme and test photograph are shown in Figure 11. The length of the metal plate is 200 cm, and the upper edge and lower edge are 80 cm and 160 cm, respectively. The sensor is located in the middle of the metal plate to measure the surface current density in *y*-direction.

**Figure 12:**
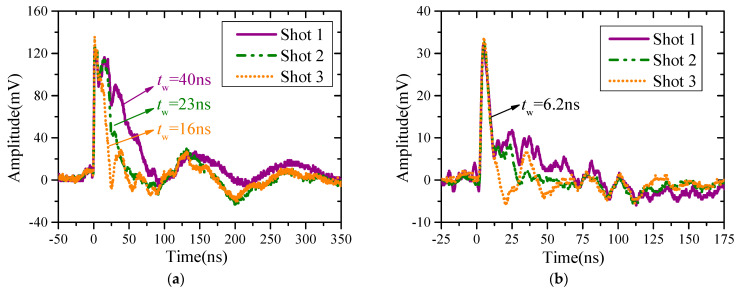
Original measured results: (**a**) ***E***-field waveforms; (**b**) surface current density waveforms.

A Marx generator can produce double exponential with rise time of 2.5 ± 0.5 ns. By adjusting the parameters of the Marx generator, three double-exponential EMP waveforms with 16 ns, 23 ns, and 40 ns half-height width, respectively, are used to irradiate the metal plates. The incident electromagnetic pulse waveforms monitored by the ***E***-field detector are shown in Figure 12a. The electric field intensity is about 7 kV·m^−1^.

The original measurement waveforms measured by the surface current density sensor are shown in Figure 12b. The measured wave characteristics are consistent with the simulated results of flat plate structure, and the peak value of the converted surface current density is 40 A/m. Under the three irradiated EMP, the surface current density waves have good consistency at the initial 12 ns, and there are some differences in the later period.

The reason for these differences is that, on one hand, there are differences in the trailing edge of the irradiated pulse. On the other hand, the larger the half-height width of EMP, the more low-frequency energy it has. The lower the frequency, the closer the metal plate is to a square in electrical size, so the process of induced current rebalancing will appear as a superimposed damped oscillation wave on a slowly fading DC component. This phenomenon is also verified in the simulation calculation.

## 5. Conclusions

Based on the theoretical and simulation analysis, the guiding basis of detector design is given for measuring the surface current density under HEMP excitation. A self-integrating surface current sensor with an optical fiber transmission link is designed to overcome the disadvantage of B-dot probe. The design process of the sensor is introduced in detail. A well-defined pulsed electromagnetic field inside a TEM cell is created; by comparing the time-domain field waveform and sensor output waveform, the sensitivity coefficient of the measurement system is 1.23 (A·m^−1^)/mV, the dynamic range is ±25~1400 A·m^−1^ (35 dB), and the bandwidth is 300 kHz~500 MHz. The surface current density of the metal plate is measured in a bounded EMP simulator, and the performance of the developed sensor is verified.

The proposed sensor is small in size and its fabrication cost is low. It can test the surface current density waveform directly without extra data processing. The adopted optical fiber transmission link has the advantages of light weight and anti-interference, which can improve the convenience of engineering applications.

The next step is optimizing the performance of the integrating circuit, especially expanding the limitation of the minimum frequency; if the −3 dB bandwidth starts at 100 kHz, the sensor can be extended to monitor the ***H***-field of the radiated HEMP in a simulator.

## Data Availability

Not applicable.
